# Naoling decoction restores cognitive function by inhibiting the neuroinflammatory network in a rat model of Alzheimer’s disease

**DOI:** 10.18632/oncotarget.17337

**Published:** 2017-04-21

**Authors:** Zian Xia, Weijun Peng, Shunhua Cheng, Bingwu Zhong, Chenxia Sheng, Chunhu Zhang, Wei Gong, Shuai Cheng, Jun Li, Zhe Wang

**Affiliations:** ^1^ Department of Integrated Traditional Chinese and Western Medicine, The Second Xiangya Hospital, Central South University, Changsha, Hunan 410011, China; ^2^ Department of Integrated Traditional Chinese and Western Medicine, Xiangya Hospital, Central South University, Changsha, Hunan 410008, China; ^3^ Department of Urology, The Second Xiangya Hospital, Central South University, Changsha, Hunan 410011, China; ^4^ Department of Neurology, Liuyang Hospital of Traditional Chinese Medicine, Liuyang, Hunan 4103002, China; ^5^ Thyroid Tumour Internal Medicine Department, Cancer Hospital Affiliated to Xiangya School of Medicine, Central South University, Changsha 410013 Hunan, China

**Keywords:** Alzheimer's disease, Naoling decoction, amyloid-beta (Aβ) deposits, neuroinflammatory network, Chromogranin A

## Abstract

Neuroinflammation is central to the pathogenesis of Alzheimer's disease (AD). We previously showed that Naoling decoction (NLD), a traditional Chinese medicine, was effective against AD, acting by inhibiting expression of IL-1β and IL-6. In the present study, we generated the rat model of AD by injecting Aβ_1–42_ peptide intracerebroventricularly and evaluated the dose-dependent effects of NLD treatment. The NLD-treated rats exhibited significant improvements in cognitive function as evaluated by the Morris water maze test. Golgi-Cox staining revealed that NLD treatment dose-dependently increased dendritic spines in the CA1 region, which were diminished in vehicle-treated rats. Further, NLD treatment normalized hippocampal Chromogranin A levels, which were elevated by Aβ_1-42_ induction. NLD also attenuated activation of microglia and astrocytes induced by Aβ_1-42_. Subsequently, NLD dose-dependently reduced levels TNF-α, IL-1β and IL-6 by inhibiting the NF-κB signaling pathway and the ASC-dependent inflammasome in the hippocampus. These findings reveal that NLD is a promising therapeutic agent that exerts inhibitory effects at multiple sites within the neuroinflammatory network induced in AD.

## INTRODUCTION

Alzheimer's disease (AD) is an irreversible neurodegenerative disease that is clinically characterized as a cognitive disorder and is the leading cause of dementia in the elderly [1]. Currently, it is one of the big healthcare challenges of the 21st century with more than 40 million people affected worldwide and its incidence is expected to substantially increase [2]. By 2050, the AD patient cohort is expected to be 115 million, costing approximately € 1.6 trillion per year [3]. Inspite of this grave situation, no effective treatments are available for AD [1] and there is an urgent need for novel effective AD therapeutics [4].

Recent evidences have indicated that neuroinflammation is central for AD pathogenesis [5–7], contributing to Aβ depositions [8], neurofibrillary tangles [9], neuronal synaptic loss and cognitive dysfunction [10, 11]. Neuroinflammation involves a complex network and includes many components like microglia, astrocytes and neurons [5]. PET analysis demonstrated that microglia cells were activated in early AD, prior to Aβ deposits [12, 13]. The activated microglia, in addition to releasing inflammatory cytokines, also activated the astrocytes thereby contributing to Aβ deposition in multiple ways [8, 14]. The Aβ deposits further exacerbate the activation and aggregation of microglia and astrocytes resulting in damage to the nearby neurons [5]. Meanwhile, the impaired neurons produce Chromogranin A (CGA) that also promotes the inflammatory response [15, 16]. Therefore, the complex, intricate interactions between microglia, astrocytes, neurons and the Aβ deposits constitutes a complex neuroinflammatory network in the AD brain. Hence, suppression of the neuroinflammation is a major therapeutic target for AD.

Thus far, several drugs like NSAIDs, pioglitazone, minocycline, and thalidomide have been tested to protect AD patients from neuroinflammation. Although these drugs have shown promise in epidemiological and basic studies, they haven't succeeded in randomized controlled trials as single targets [5]. In this background, traditional Chinese medicine (TCM) is becoming increasingly accepted as novel therapeutics. They have been used in traditional clinical practices for many centuries and are composed of numerous active compounds that exert multi-target effects [17]. Naoling decoction (NLD) is one such TCM formula that has been used to treat AD [18–21]. It is derived from an ancient formula of Kongsheng Zhenzhong Dan, first described in *Qianjin Yaofang*, a book written by Simiao Sun of the Tan Dynasty nearly 1364 years ago. NLD consists of 6 crude herbs, namely, *Herba epimedii (Yinyanghuo)*, *Polygonum multiflorum (Heshouwu)*, *Tortoise plastron (Guiban)*, *Ossa draconis (Longgu)*, *Polygala tenuifolia (Yuanzhi)*, and *Rhizoma acori graminei* (Shichangpu). Our previous studies showed that NLD reduces the production of inflammatory cytokines in both the AD patients and the rat AD model [18, 19]. Although several NLD-derived compounds, such as 2,3,5,4′-tetrahydroxystilbene-2-O-β-D-glucopyranoside (THSG) and Icariin, have been shown to possess an ability to alleviate inflammatory response [22–25], the detail mechanisms by which the NLD mitigates neuroinflammation during AD are unclear.

Therefore, in the present study, we used the experimental rat AD model by intra-cerebroventricular injection of Aβ_1–42_, determined the efficacy of NLD in attenuating activation of microglia and astrocyte and evaluated the effects on cognitive impairment due to neuronal damage in the hippocampus. Furthermore, we explored the effects of NLD on NF-κB signaling pathway, ASC/caspase-1/IL-1β axis and CGA release that are mechanistically important for AD pathology.

## RESULTS

### Molecular characterization of NLD using liquid chromatography-mass spectrometry quadrapole time of flight (LC/MS-Q-TOF)

**Figure 1:**
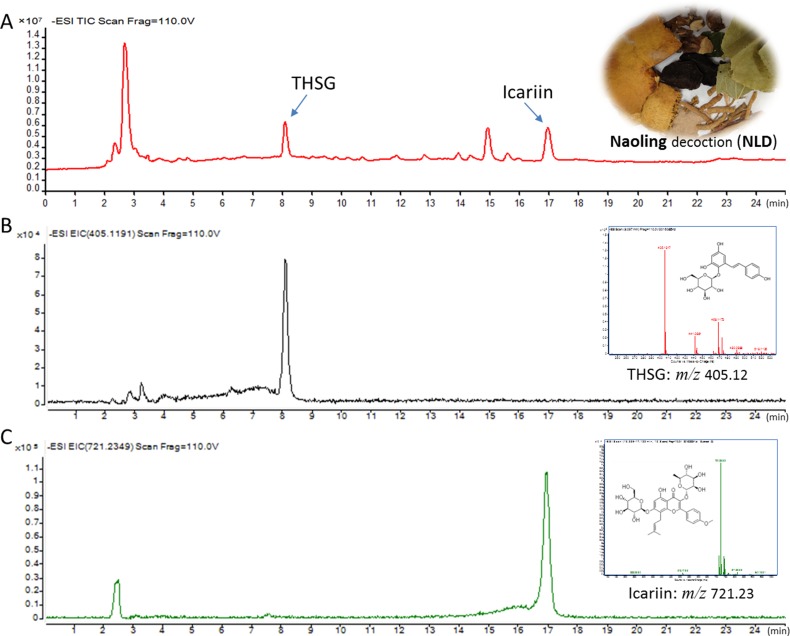
Liquid chromatography-mass spectrometry quadrapole time of flight (LC/MS-Q-TOF) analysis of Icariin and 2,3,5,4′-tetrahydroxystilbene-2-O-β-D-glucopyranoside (THSG) (**A**) LC/MS-Q-TOF TIC chromatograms of NLD in the negative ESI mode. (**B**) LC/MS-Q-TOF EIC chromatograms of THSG in the negative ESI mode showing m/z ratio as 405.12 (top right graph). (**C**) LC/MS-Q-TOF EIC chromatograms of icariin in the negative ESI mode showing m/z ratio as 721.23 (top right graph).

THSG and Icariin, which are constituents of NLD were selected as quality control standards in our study [17]. The quantitative and qualitative analyses of THSG and Icariin were conducted using the LC/MS-Q-TOF in a negative ESI mode using 0.1% methanol in water and acetonitrile for chromatogram separation (Figure 1). At the beginning, retention time and MS were conducted according to the analyses of standard substances. The retention times of THSG and Icariin were 8.09 ± 0.03 min and 16.95 ± 0.05 min, respectively (Figure 1B–1C). The mass/charge (m/z) ratio in the MS for TSHG and Icariin were 405.12 and 721.23, respectively (Figure 1B–1C). Subsequently, the EIC chromatograms were used to determine the contents of NLD and the m/z ratios for TSHG and Icariin were 405.1191 and 721.2349, respectively (Figure 1B–1C). Their intra- and inter-day variations were less than 5%. More than 90% of the two compounds were recovered under these test conditions. Thus, the method was validated and found to be efficient. The results showed that TSHG and Icariin constituted 1.206 ± 0.081 mg and 0.738 ± 0.043 mg per gram of NLD, respectively.

### NLD improved the cognitive recovery after intra-cerebroventricular injection of Aβ_1-42_

**Figure 2:**
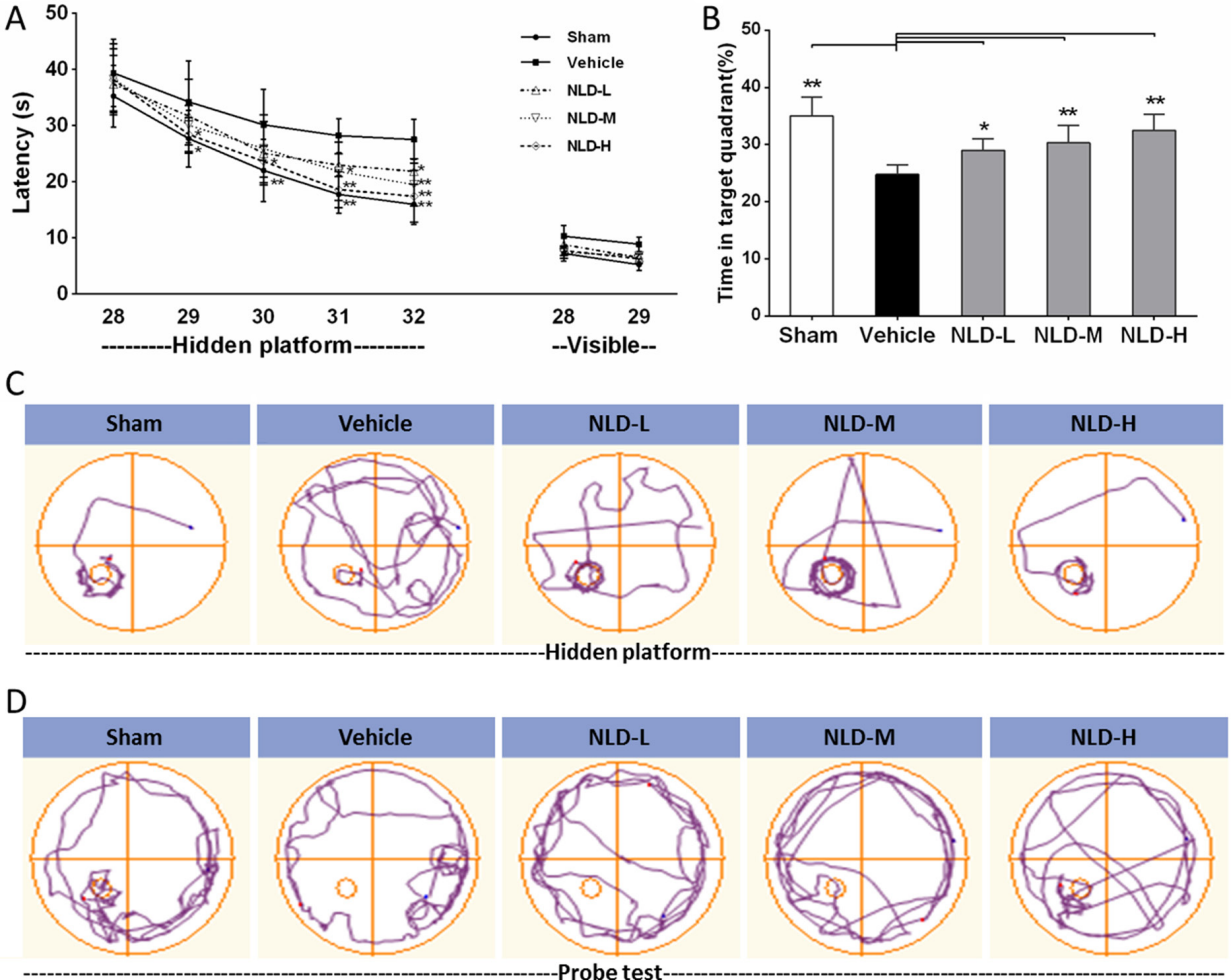
Effect of NLD on cognitive outcomes in Aβ_1-42_-infused rat model (**A**) Rats that were intracerebroventricularly injected with Aβ_1-42_ were administered NLD (NLD-L group: 9 g/kg; NLD-M group: 27 g/kg; NLD-H group: 54 g/kg) or an equivalent amount of saline (vehicle group) by oral gavage each day. Meanwhile, sham group rats received equal amount of saline orally. The Morris water maze (MWM) test was performed on all groups of rats to evaluate the cognitive function from the 28th to the 32nd day. The NLD group rats demonstrated significant improvements in the hidden platform tests compared to vehicle group rats (*p* < 0.01 for the group) and learning time. (**B**) The probe tests on the 32nd day showed that NLD treated rats showed significantly longer residence time in the target quadrant compared with the vehicle group (all *p* < 0.05). No differences in visible platform performance were noted (*n* = 8/group). (**C**) Representative images of the swim paths of different rat groups in the hidden platform testing on the 32nd day. (**D**) Representative images of the swim paths of different rat groups in the probe test. The data of escape latency was analyzed by two-way ANOVA, whereas the data from probe test were analyzed by one-way ANOVA. All data are presented as the mean ± SEM. *p < 0.05, **p < 0.01 compared to the vehicle group.

Next, we assessed the effects of NLD on the cognitive function in AD rats by testing their ability to escape to a hidden platform in the Morris water maze (MWM) test conducted from 28th to 32nd day after Aβ_1-42_infusion. The escape latency in the hidden platform training was used to evaluate the spatial learning of 5 different groups of rats. First, we observed that the vehicle group rats significantly increased escape latency than the Sham group rats from the 29th to 32nd day (Figure 2A). This indicated that the intra-cerebroventricular injection of Aβ_1-42_ impaired the spatial learning ability of the rats. Further, all the 3 groups of NLD rats showed reduced escape latency compared to the vehicle group rats (Figure 2A). Interestingly, there was dose-dependent effect of NLD on escape latency, with the NLD-H group rats (54 g/kg/day) demonstrating improved ability on days 29 to 32, whereas the NLD-M group rats (27 g/kg/day) demonstrated improved ability on days 31 and 32 and the NLD-L group rats (9 g/kg/day) demonstrated improved escape latency only on day 32 (Figure 2A–2B). This demonstrated that NLD ameliorated the impaired ability of spatial learning in AD in a dose-dependent manner. Also, in visible test, we did not observe significant differences among each group of rats suggesting that the cognitive impairment from Aβ_1-42_ injection uniformly influenced the cognitive function data from the MWM tests.

After the hidden platform training on the 32nd day, a probe test was conducted to estimate spatial memory ability by calculating the time spent by the rats of different groups in the target quadrant (Figure 2B). We observed that the vehicle group rats spent significantly shorter time in the target quadrant compared to the Sham group rats (*p* < 0.01). Also, the three groups of NLD treated rats showed significantly increased residence time than the vehicle group (*p* < 0.05) in the target quadrant. This showed that NLD significantly improved the spatial memory that was impaired due to AD. Further, the higher NLD doses demonstrated better cognitive recovery, though not statistically significant (Figure 2A and 2B).

### NLD significantly enhanced the dendritic spines of neuron and reduced the CGA release in hippocampus of Aβ_1-42_-infused rat

**Figure 3:**
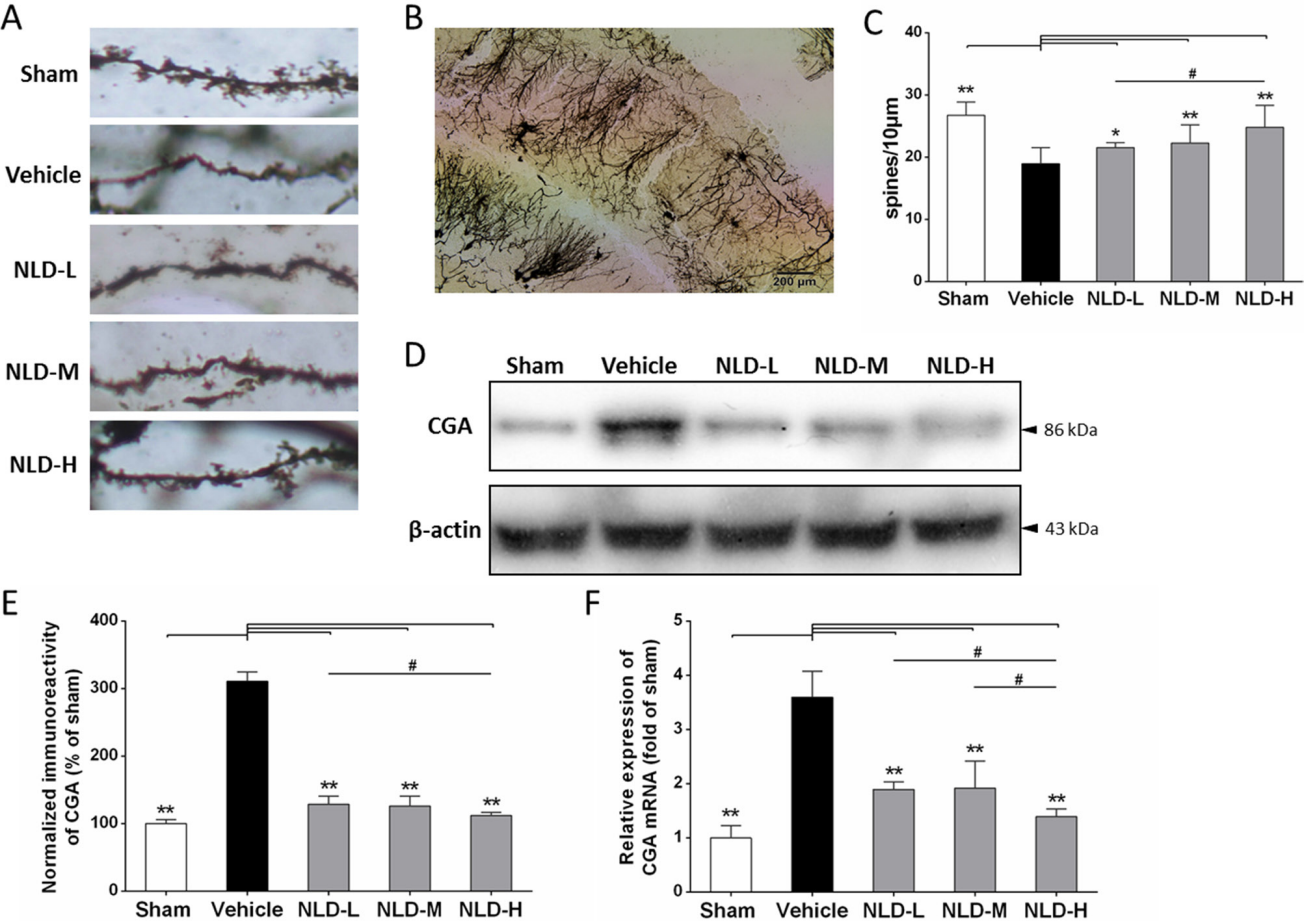
Effects of NLD on dendritic spines of neuron and release of Chromogranin A (CGA) in Aβ_1-42_-infused rat model (**A**) The representative images of Golgi-Cox stained dendritic spines in sham, vehicle, NLD-L, NLD-M and NLD-H rat groups *n* = 3/group). (**B**) A representative image of Golgi-Cox staining of the hippocampal CA1 subregion in vehicle rat group infused with Aβ_1-42_ only. (**C**) The dendritic spine density in the 5 rat groups. The pyramidal neurons with countable morphology were selected from CA1 subregion (sham: *n* = 25, vehicle: n = 18, NLD-L: *n* = 21, NLD-M: *n* = 23, and NLD-H: *n* = 20) and the spine numbers were counted in three to four dendrites per neuron (dendritic spine density = spine numbers/dendritic length). Rats from vehicle group showed significantly reduced the dendritic spine density compared to the sham group (vehicle group: 18.95 ± 2.52 spines/10 μm, *n* = 18; sham group: 26.74 ± 2.18 spines/10 μm, *n* = 25; *p* < 0.01). Rats treated with NLD showed dose-dependent enhancement in dendritic spine density compared with the vehicle group (NLD-L group: 21.06 ± 0.79 spines/10 μm, *n* = 21; NLD-M group: 22.28 ± 1.95 spines/10 μm, *n* = 23; NLD-H group: 24.83 ± 1.54 spines/10 μm, *n* = 20; all *p* < 0.05). (**D**) Representative western blot image of CGA expression in hippocampus from the 5 rat groups. (**E**) Quantitative analysis of hippocampal CGA in different groups. The data are represented as the percentage of sham group (n = 6/group). (**F**) The qRT-PCR analysis of CGA mRNA in the hippocampus from different groups. Data are presented relative to the sham group (*n* = 6/group). NLD groups demonstrate decreased CGA protein and mRNA levels in hippocampus compared with the vehicle group. Significant differences in NLD-H versus NLD-L/NLD-M are also noted. The data were analyzed by one-way ANOVA and presented as the mean ± SEM. *p < 0.05, **p < 0.01 vs. the vehicle group. ^#^*p* < 0.05 vs. the NLD-H group.

Next, we evaluated the status of synaptic failure during AD by estimating the dendritic spine density of the CA1 subregion of the rat brain by Golgi-Cox staining of the rat brains from each experimental group as described in previous study [26]. As shown in Figure 3C, the rats from the vehicle group had lower dendritic spine density compared to the sham group (vehicle group: 18.95 ± 2.52 spines/10 μm, *n* = 18; sham group: 26.74 ± 2.18 spines/10 μm, *n* = 25; *p <* 0.01). Treatment with NLD significantly enhanced the dendritic spine density in a dose-dependent manner, in comparison to the vehicle group (NLD-L group: 21.06 ± 0.79 spines/ 10 μm, *n* = 20; NLD-M group: 22.28 ± 1.95 spines/10 μm, *n* = 23; NLD-H group: 24.83 ± 1.54 spines/10 μm, *n* = 21; *p <* 0.05). The NLD-H group had significantly higher number of dendritic spines than the NLD-L group (*p* < 0.05). These data demonstrated that NLD repaired the failure of synapse induced by the Aβ deposits during AD. Concurrently, marked increase in CGA protein and mRNA levels were observed in the vehicle group relative to the sham group (*p* < 0.01), whereas the NLD groups had significantly lower CGA protein and mRNA compared to the vehicle group (*p* < 0.05; Figure 3D–3F). Importantly, NLD-H group of rats had significantly lower CGA protein and mRNA than NLD-L rats (*p* < 0.05) and lower CGA mRNA expression relative to the NLD-M rats. These data suggested that NLD suppressed the release of CGA in the hippocampus due to Aβ deposits in a dose-dependent manner.

### NLD inhibited the activation of microglia and astrocytes in the hippocampus after intracerebroventricular injection of Aβ_1-42_

**Figure 4:**
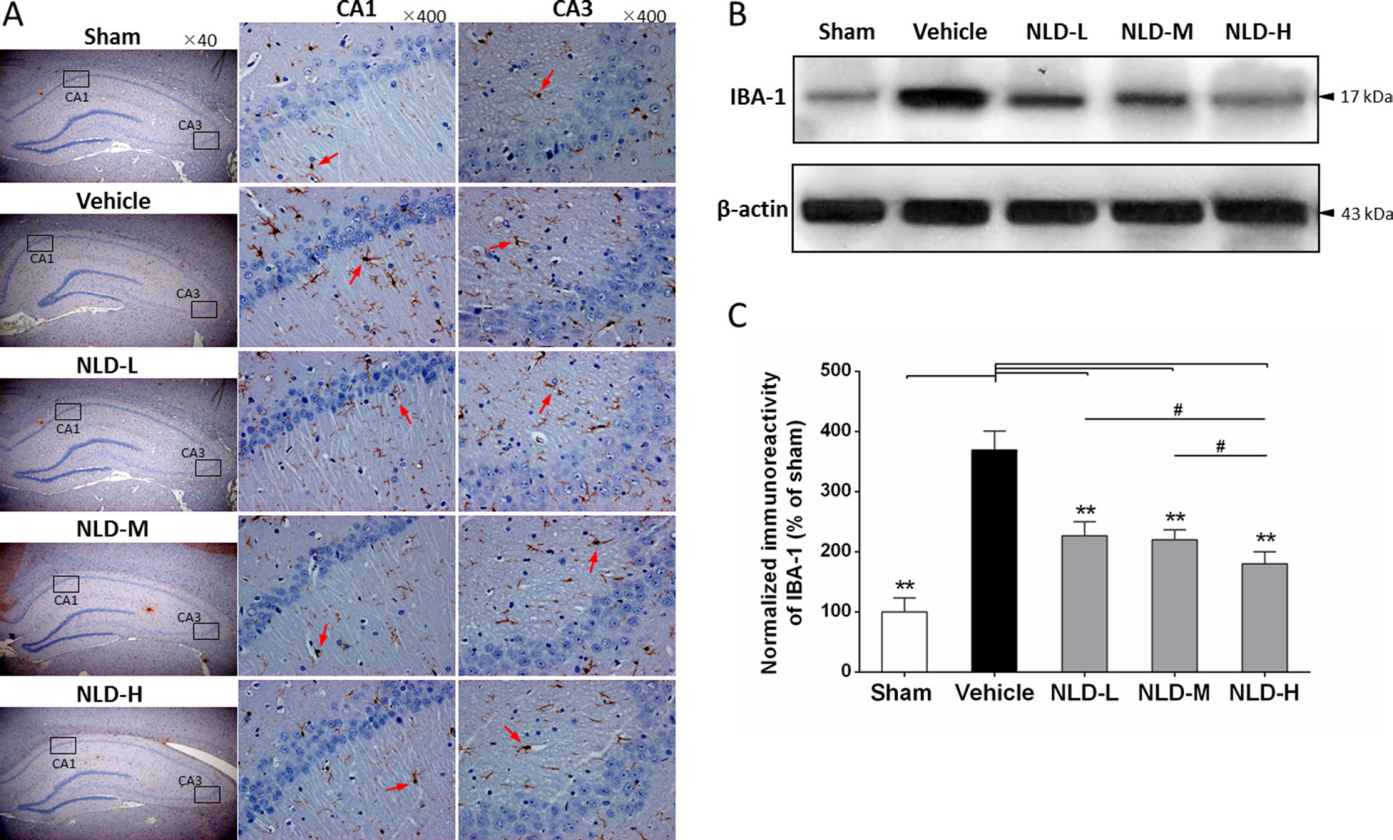
NLD inhibits microglial activation in hippocampus after intracerebroventricular injection of Aβ_1-42_ (**A**) The representative immunohistochemistry images of IBA-1 in sham, vehicle, NLD-L, NLD-M, and NLD-H groups (*n* = 3/group). The images from the first column show the total hippocampus (40× magnification). The selected CA1 and CA3 regions using black frames were enlarged 10 times and shown in the second and third column, respectively. The red arrows indicate the activated microglia. (**B**) A representative western blot image of IBA-1 expression in hippocampus from different groups. (**C**) Quantitative analysis of IBA-1 in hippocampus from each group. The data are represented as the relative percentage of sham group (*n* = 6/group). All the NLD groups significantly reduced the upregulation of IBA-1 induced by Aβ_1-42_infusion. Marked differences between NLD groups (NLD-L and NLD-M vs. NLD-H) were also observed. All data were analyzed by one-way ANOVA and presented as the mean ± SEM. *p < 0.05, **p < 0.01 vs. the vehicle group. ^#^*p* < 0.05 vs. the NLD-H group.

Since one of characteristics of AD pathology is activation and aggregation of microglia and astrocytes induced by Aβ deposits [5], we analyzed markers for microglial and astrocyte activation namely IBA-1 and GFAP by immunohistochemistry and western blot. As shown in Figure 4A, IHC analysis of IBA1 demonstrated increased number of activated microglia in the CA1/CA3 region from the vehicle group relative to the sham group, whereas the NLD treatment groups showed reduced number of activated microglia compared to the vehicle group. Further quantitative western blot analyses of IBA-1 was consistent with the IHC data and showed that all the NLD treatment groups inhibited IBA-1 overexpression compared to the vehicle group (*p <* 0.01; Figure 4B–4C). In addition, downregulation of IBA-1 in the NLD-H group was more pronounced than the NLD-L and NLD-M groups (both *p* < 0.05). These results suggested that NLD treatment significantly inhibited activation of microglia in the hippocampus due to intra-cerebroventricular injection of Aβ_1-42_ in a dose-dependent manner.

**Figure 5:**
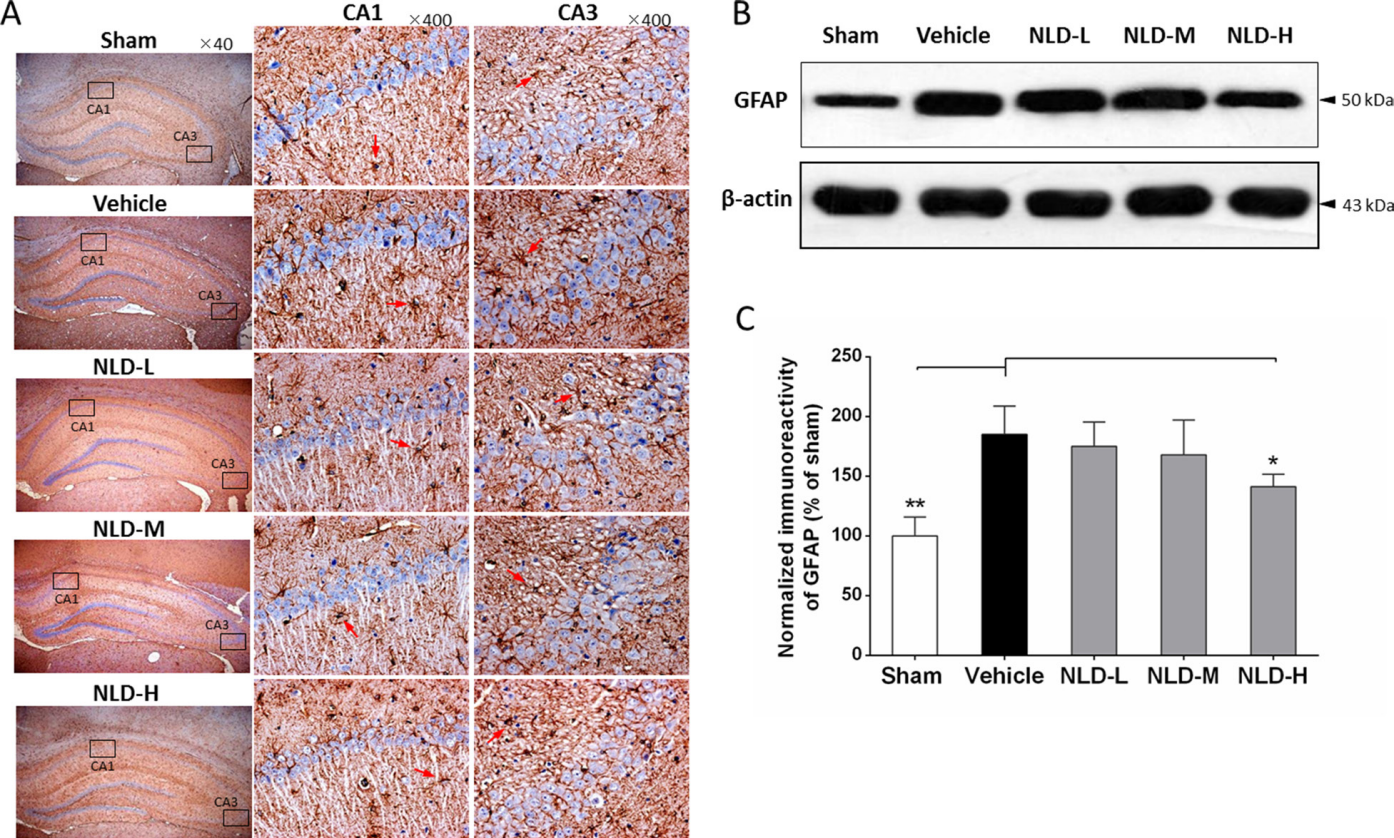
NLD inhibits astrocyte activation in hippocampus after intracerebroventricular injection of Aβ_1-42_ (**A**) The representative immunohistochemistry images of GFAP in sham, vehicle, NLD-L, NLD-M, and NLD-H groups (*n* = 3/group). The images from first column showed the total hippocampus (40 × magnification). The selected CA1 and CA3 regions using black frames were enlarged 10 times and exhibited in the second and third column, respectively. The red arrows indicate the activated astrocytes. (**B**) A representative western blot image of GFAP expression in hippocampus from different groups. (**C**) Quantitative analysis of GFAP in hippocampus from different groups. The data are represented as the relative percentage to the sham group (*n* = 6/group). Only NLD-H group significantly reduced the upregulation of GFAP after Aβ_1-42_ infusion. The data were analyzed by one-way ANOVA and are presented as the mean ± SEM. *p < 0.05, **p < 0.01 vs. the vehicle group.

Similarly, GFAP immunohistochemistry showed higher number of activated astrocytes in the vehicle group compared to the sham group and decreased number of activated astrocytes in all the NLD treatment groups (Figure 5A). The western blot analyses of GFAP also showed that the vehicle group had enhanced GFAP levels compared to the sham group (*p* < 0.01; Figure 5B–5C). Further, significant reduction in GFAP was observed in NLD-H group (*p* < 0.05; Figure 5B–5C). In the NLD-L and NLD-M groups, although GFAP levels tended to be low, they were not statistically significant. These data demonstrated that NLD was capable of preventing the activation and aggregation of astrocytes in the hippocampus following Aβ deposits in the rat AD brains.

### NLD notably attenuated the production of pro-inflammatory cytokines as well as their mRNA transcription in hippocampus after intracerebroventricular injection of Aβ_1-42_

**Figure 6:**
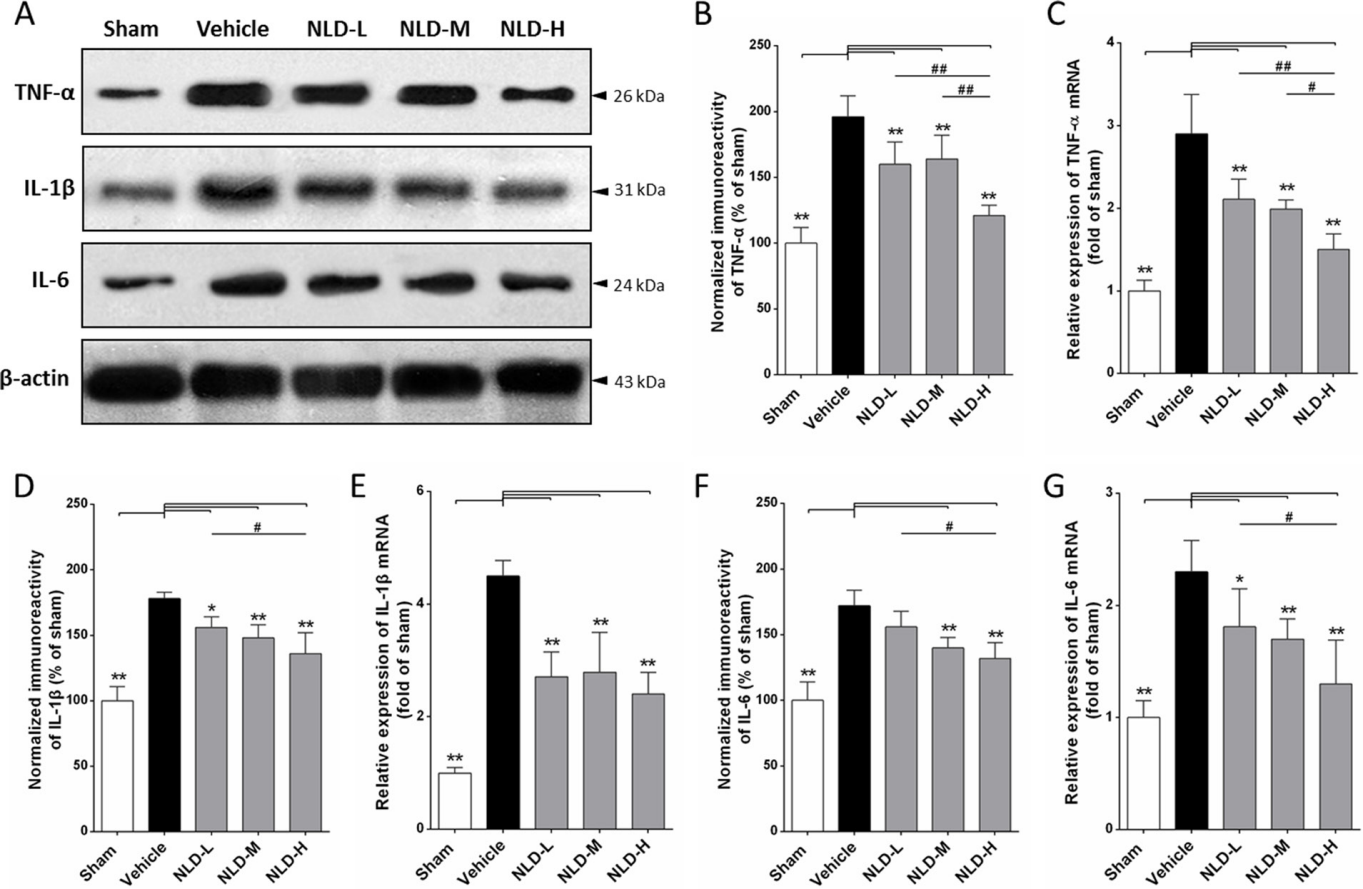
NLD inhibits expression of pro-inflammatory cytokines TNF-α, IL-1β and IL-6 in the hippocampus after intracerebroventricular injection of Aβ_1-42_ (**A**) Representative western blot images of TNF-α, IL-1β and IL-6 expression in hippocampus from different groups. (**B**, **D**, **F**) The expression levels of TNF-α, IL-1β and IL-6 proteins in the 5 groups of rats. (**C**, **E**, **G**) The expression levels of TNF-α, IL-1β and IL-6 mRNA in the 5 groups of rats. Aβ_1-42_ injection into the paracele significantly increased levels of TNF-α, IL-1β, IL-6 proteins and mRNA in the hippocampus of vehicle rats. NLD groups significantly reduced the increased protein and mRNA levels of TNF-α, IL-1β and IL-6, except for the IL-6 protein levels in the NLD-L group. There were significant differences between NLD-L group and NLD-H group in TNF-α, IL-1β and IL-6 protein and TNF-α and IL-6 mRNA. Also, significant differences between NLD-M group and NLD-H group were observed in the protein and mRNA expression of TNF-α. The data were analyzed by one-way ANOVA and are presented as the mean ± SEM. *p < 0.05, **p < 0.01 vs. the vehicle group. ^#^*p* < 0.05, ^##^*p* < 0.01 vs. the NLD-H group.

Further, we assessed inflammation in the hippocampus by quantitating the protein and mRNA levels of pro-inflammatory cytokines, TNF-α, IL-1β and IL-6 by western blot and qRT-PCR, respectively. We observed that the Aβ_1-42_ injection into bilateral cerebral ventricle (vehicle group) significantly increased the TNF-α, IL-1β and IL-6 protein and mRNA levels in comparison to the sham group (*p* < 0.01 vs. Sham group, Figure 6B, 6D and 6F). NLD treatment significantly reduced the TNF-α protein and mRNA levels in a dose-dependent manner (all *p <* 0.01 vs. vehicle group, Figure 6B–6C). Further, we observed that NLD treatment significantly reduced IL-1β protein and mRNA levels that were increased in the vehicle group in a dose-dependent manner (Figure 6D–6E). We noted that the statistical significance was also evident between the NLD-H and NLD-L in terms of IL-1β protein levels (Figure 6D). Further, the NLD-M and NLD-H group rats showed significantly decreased IL-6 protein levels and all the 3 NLD groups of rats demonstrated significantly decreased IL-6 mRNA compared to the vehicle group (Figure 6F). These results indicated that NLD treatment significantly suppressed inflammation in a dose dependent manner.

### NLD inhibited the both activated NF-κB signaling pathway and ASC-depending inflammasome in the hippocampus after intracerebroventricular injection of Aβ_1-42_

**Figure 7:**
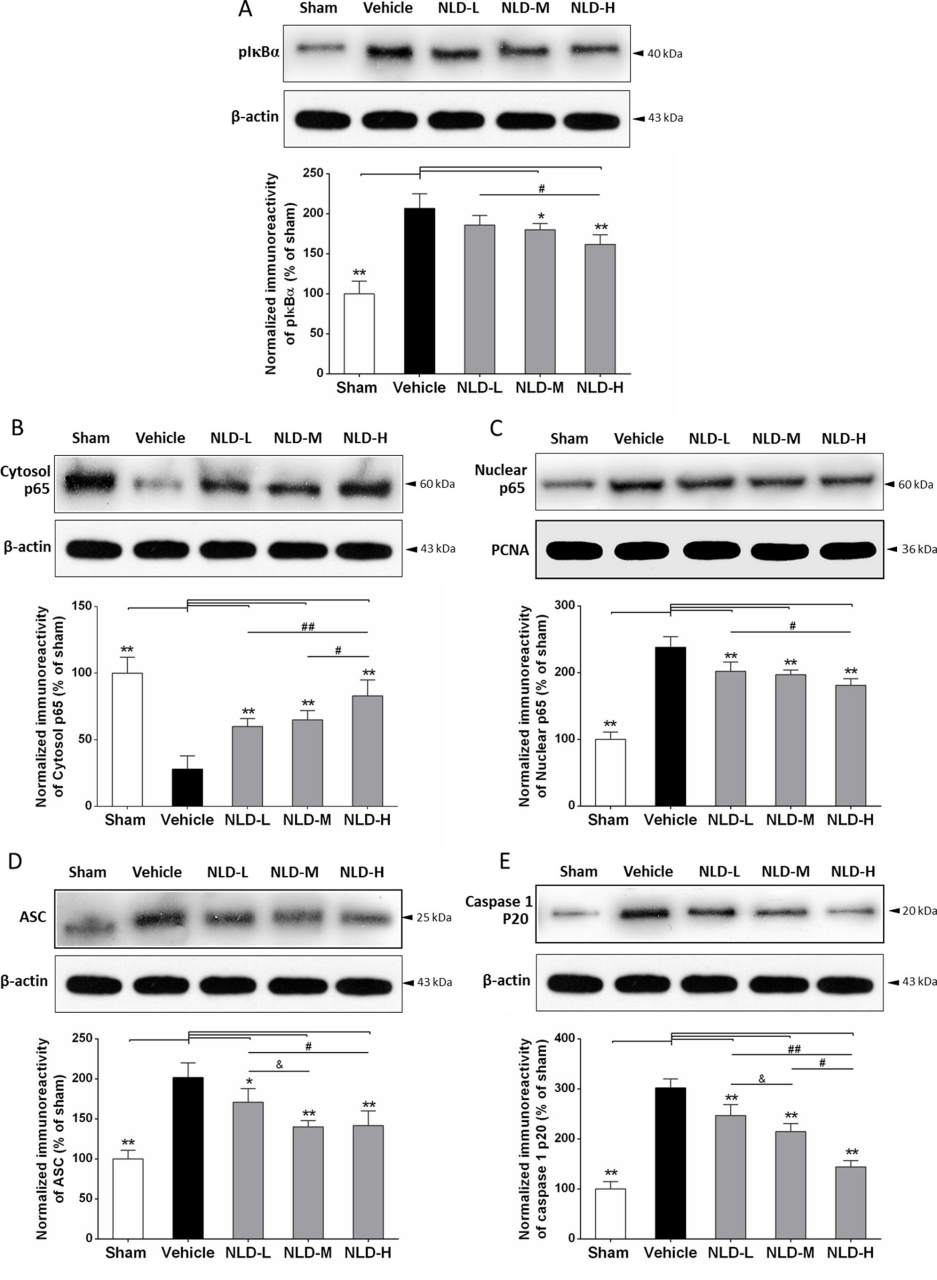
NLD inhibits the NF-κB signaling pathway and ASC-depending inflammasome in the hippocampus after intracerebroventricular injection of Aβ_1-42_ Representative western blots and quantitative analysis of the effects of NLD treatment on (**A**) pIκBα. (**B**) cytoplasmic p65. (**C**) nuclear p65 (**D**) ASC and (**E**) caspase1 p20 proteins in the hippocampus on the 32nd day after Aβ_1-42_ infusion. The expression of pIκBα, nuclear p65, ASC and caspase 1 p20 were increased markedly in vehicle group compared with the sham group, and significantly decreased in the NLD groups except for pIκBα expression in the NLD-L group. On the contrary, the cytoplasmic p65 was markedly reduced in vehicle group compared with the sham group, whereas NLD groups showed significantly higher cytoplasmic p65 than the vehicle group. Significant differences between NLD-H and NLD-L group were also observed in pIκBα, cytoplasmic/nuclear p65, ASC and caspase1 p20 levels. There were significant differences in cytoplasmic p65 and caspase1 p20 levels between NLD-M and NLD-H groups, as well as ASC and caspase1 p20 levels between NLD-M and NLD-L groups. PCNA and β-actin were used as controls for nuclear and cytoplasmic p65, respectively. The quantitative data is represented as the relative percentage to the sham group (*n* = 6/group). The data were analyzed by one-way ANOVA and are presented as the mean ± SEM. *p < 0.05, **p < 0.01 vs. the vehicle group. ^#^*p* < 0.05, ^##^*p* < 0.01 vs. the NLD-H group. & *p* < 0.05 NLD-H vs. NLD-M group.

We further studied the effect of NLD on the status of the NF-κB signaling pathway and ASC-dependent inflammasome that are critical for inflammation induced during AD by evaluating pIκBα, cytoplasmic/nuclear p65, and ASC and caspase-1 p20 in the hippocampus by western blot. We observed that intracerebroventricular injection of Aβ_1-42_ resulted in significant upregulation of pIκBα, nuclear p65, ASC and caspase-1 p20 proteins in the vehicle group rat hippocampus compared to the sham group (*p <* 0.01; Figure 7A, 7B, 7D and 7E) and decreased cytoplasmic p65 (*p <* 0.01; Figure 7C). These data suggested that Aβ deposits in the brain activate the NF-κB signaling pathway and ASC-dependent inflammasome formation in the hippocampus. Further, except for no significant reduction for pIκBα in the NLD-L group, other NLD treatments demonstrated significantly low pIκBα, nuclear p65, ASC and caspase-1 p20 levels compared to the vehicle group (*p <* 0.05; Figure 7A, 7B, 7D, 7E) and enhanced cytoplasmic p65 (*p <* 0.01; Figure 7C). The NLD-H group of rats demonstrated the most pronounced effects. Interestingly, NLD-M that had no significant difference in pIκBα and cytoplasmic/nuclear p65 expression compared to the NLD-L group showed stronger reduction in ASC and caspase-1 p20 than NLD-L (*p <* 0.05; Figure 7D, 7E). These results suggested that NLD treatment inhibited the activated NF-κB signaling pathway and formation of ASC-dependent inflammasome in the hippocampus in a dose-dependent manner.

## DISCUSSION

This is the first study in our knowledge to have investigated the effects of TCM treatment on neuroinflammatory network in the AD brain. Our study in the AD rat model demonstrates that NLD has significant benefits. We demonstrate improved cognition as a result of increased dendritic spines in the CA1 region and suppressed microglial and astrocyte activation upon intracerebroventricular injection of Aβ_1-42_. Further, we demonstrate reduced CGA, pro-inflammatory cytokines (i.e. TNF-α, IL-1β and IL-6), NF-κB signaling pathway activation and ASC induced inflammasome in the rat hippocampal region. These results suggest that the NLD is neuroprotective against AD by suppressing the neuroinflammatory network between microglia, astrocyte and neurons that are involved in activation of the NF-κB pathway and the ASC-dependent inflammasome/caspase-1/IL-1β axis.

In AD, the Aβ deposits activate microglia [12], which are key for AD pathology [6]. In a controlled environment, activated microglia produce anti-inflammatory cytokines for tissue repair and growth by upregulating neuroimmune regulatory proteins (NiRegs) [27]. However, persistent stimulation by the accumulated Aβ deposits results in microglial recruitment to the site of the local lesion [28] and chronic activation induced by excessive cytokines [6, 29]. This results in an inflammatory storm that further releases pro-inflammatory factors, thereby facilitating Aβ deposition with ineffective phagocytes that aggravates cognitive impairment by stripping synapses from the neurons resulting in neuronal loss [6]. In our study we demonstrated significant activation of microglia demonstrated by high IBA-1 staining in the AD rat brain (Figure 4), with concomitant production of TNF-α, IL-1β and IL-6 (Figure 6). The resultant decline in cognitive function was assessed by the MWM maze test (Figure 2) and the reduced dentritic spines were evaluated by golgi-cox staining (Figure 3A–3C). Most importantly, we demonstrated that treatment with NLD inhibited all the pathological changes due to AD including improved cognition (Figure 2). This suggested that NLD was neuroprotective against AD by mitigating both neuroinflammation and the corresponding synapse losses in the brain, thus supporting our previous findings [19].

Moreover, reactive astrogliosis is also an important morphological characteristic of AD [5]. Following AD, overproduction of proinflammatory cytokines (e.g. TNF-α and IL-1β) activates astrocytes through the TNF receptor 1 (TNFR1) or IL-1 type I receptor (IL-1RI), especially in the nearby Aβ plaques [30]. These activated astrocytes contribute to prolonging neuroinflammatory response by constantly releasing proinflammatory factors, chemokines, nitric oxide/reactive oxygen species, thereby inducing neurotoxicity [30, 31]. Consequently, the dysfunctional astrocytes result in neurodegeneration and cognitive impairment [30, 32]. Consistent with the prior studies, we demonstrated increase in activated astrocytes based on enhanced GFAP, elevated proinflammatory cytokines (TNF-α, IL-1β and IL-6) and impaired cognitive function. Further, NLD treatments diminished the number of activated astrocytes and the proinflammatory cytokines in the hippocampus and improved cognition in the rat AD model. Therefore, inhibiting astrocyte activation improved cognitive function as previously reported [33].

The accumulation of intracellular Aβ deposition in the AD brain triggers neuronal injury and synaptic damage, thereby inducing progressive neuritic dystrophy. These pathological processes result in the overproduction of CGA in the large dystrophic neurites near the plaques [16]. CGA is an acidic glycophosphoprotein, which is released with other hormones by exocytosis and contributes to neuroinflammatory progression in AD [15, 16, 34]. Excessive CGA potentiates microglial activation and the release of proinflammatory cytokines by binding to class A scavenger receptor (SRA) and Toll-like receptor 4 (TLR4) [15]. Further, proteomic studies in AD patients have shown that CGA is a biomarker of cerebrospinal fluid (CSF) for predicting the AD in early phase and denoting progression of AD [35, 36]. In the present study, we found significantly elevated CGA expression (Figure 3D–3F), activated microglia (Figure 4) and pro-inflammatory cytokines (Figure 6) in the rat AD model (vehicle group) compared to the sham group, similar to those reported in AD patients [16]. Most importantly, NLD treatment alleviated the microglial cell activation by downregulating CGA expression in the AD brain suggesting that it protects neurons in AD brains (Figure 3A–3C).

Our data also demonstrates that co-ordinated regulation of multiple pathological processes in AD opens up avenues for efficient treatment or prevention. There is significant evidence that the NF-κB signaling pathway and the inflammasome/caspase-1/IL-1β axis promote the inflammatory cascade in the AD brain [15, 37, 38], which exists in microglia, astrocytes and neurons [30, 37, 39, 40]. Aβ stimulation induces phosphorylation of IκBα and separation of the NF-κB dimmers resulting in translocation of p65 into the nucleus to initiate transcription of pro-inflammatory genes [30] and other critical factors like NLRP3 [41]. Meanwhile, the ASC-dependent inflammasome is also activated by the Aβ deposits [37, 39], which includes the NLRP3 inflammasome in microglia, IPAF inflammasome in the astrocytes and NLRP1 inflammasome in the neurons [6, 30, 39, 42]. These result in the mature IL-1β by cleaving pro-caspase-1 into caspase-1 [43]. Importantly, CGA more strongly activates both the NF-κB pathway and NLRP3 inflammasome in the microglial cells than the Aβ deposits [15]. In our study, we demonstrate activation of the NF-κB signaling pathway by showing significant upregulation of intranuclear NF-κB p65, phosphorylated IκBα and enhanced TNF-α/IL-1β/IL-6 in addition reduced cytosolic p65 in the hippocampus of the rat AD model (Figures 6, 7). In addition, we showed increased expression of ASC, cleaved caspase-1 and IL-1β (Figures 6, 7), which further demonstrated an activated ASC-dependent inflammasome/caspase-1/IL-1β axis. It has been previously demonstrated that microglia are the first responders to neuroinflammation that sense the pathological changes due to Aβ deposits through TLR4/NLRP3 receptors [44, 45] and CGA through SRA /TLR4 receptors [15]. Collectively, these activated receptors induce the activation of NF-κB signaling pathway and the NLRP3 inflammasome/caspase-1/IL-1β axis [15, 44, 45], which results in the abundant secretion of proinflammatory cytokines (TNF-α, IL-1β and IL-6). Moreover, the TNF-α and IL-1β that are released from the microglia stimulates the TNFR1 and IL1RI receptors of astrocytes, respectively, thereby activating the NF-κB pathway in the astrocytes [30]. At the same time, the Aβ deposits also activate the astrocytic IPAF inflammasome [46]. Consequently, there is further release of TNF-α, IL-1β and IL-6 from the astrocytes that augments the neuroinflammatory cascades in AD brain. Furthermore, in the neurons, the activated NLRP1 inflammasome [39] and the NF-κB signaling pathway stimulated by the Aβ deposits through the cysteinyl leukotriene receptor 1 (CysLT1R) promote the synthesis of pro-inflammatory cytokines like TNF-α and IL-1β [40]. In our study, NLD significantly inhibited both the signaling pathways (Figure 7) and therefore led to a concomitant decrease in TNF-α, IL-1β and IL-6 levels (Figure 6). These results support previous findings that demonstrate downregulation of pro-inflammatory cytokines by inhibiting the NF-κB signaling and the inflammasome/caspase-1/IL-1β axis in the CNS [24, 41]. Also, in a previous study icariin, a compound derived from NLD, inhibited both the pathways [24], thereby providing further evidence for neuroprotective effect of NLD against AD neuroinflammation.

**Figure 8:**
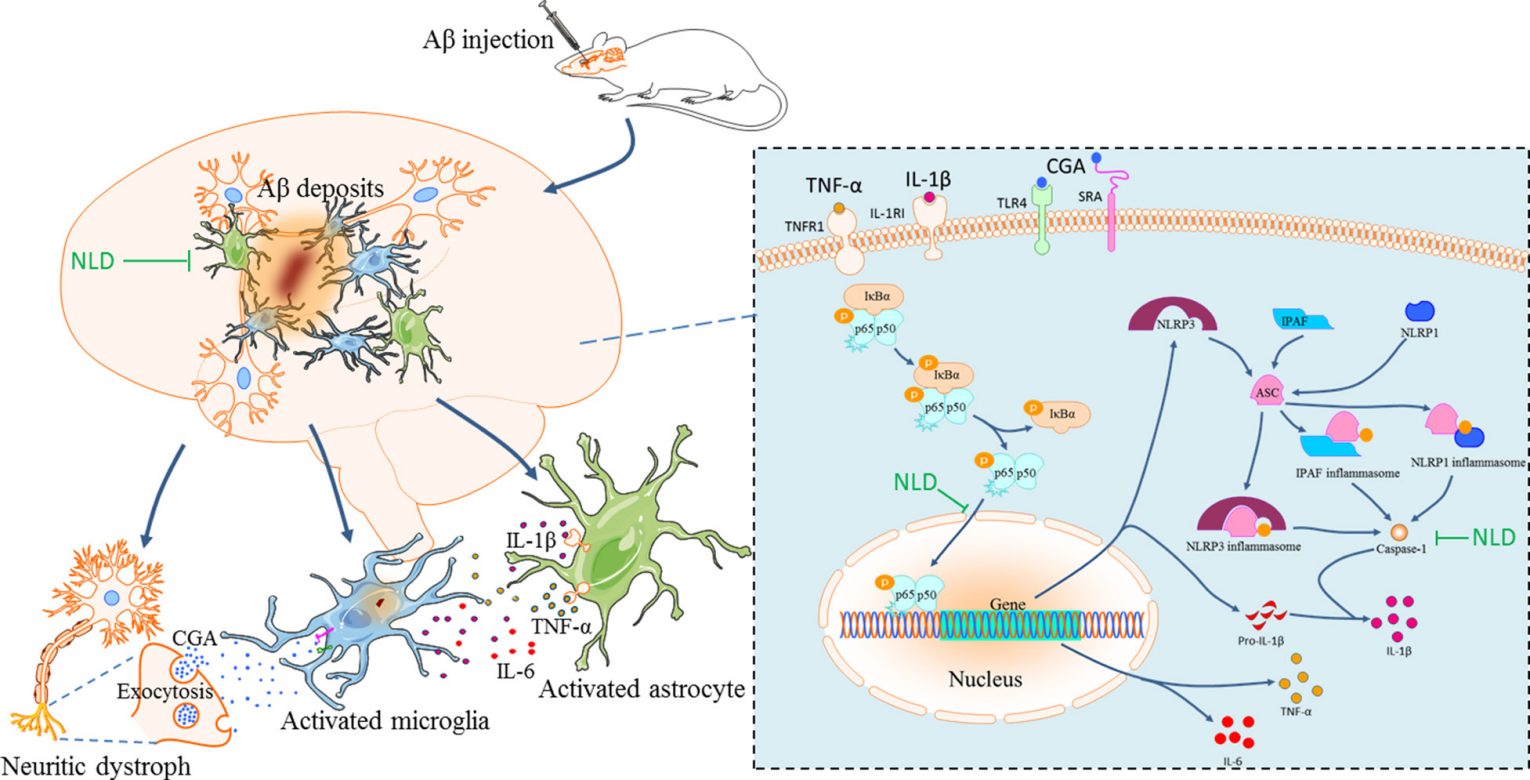
The pathological inflammatory network in the AD brain and the anti-inflammatory effects of NLD During AD, the formation of Aβ deposits not only stimulates the activation and aggregation of microglia and astrocytes, but also triggers the neuronal injury and synaptic damage, which induces a progressive neuritic dystrophy. This dystrophy of the neurons results in the over-production of Chromogranin A (CGA), which is released by exocytosis of secretory granules. CGA potentiates the microglial activation and the release of pro-inflammatory cytokines ( TNF-α, IL-1β and IL-6) by binding to class A scavenger receptor (SRA) and Toll-like receptor 4 (TLR4). The astrocytes surrounding the Aβ deposits sense the TNF-α and IL-1β by TNF receptor 1 (TNFR1) and IL1 type I receptor (IL1RI), respectively, which leads to prolonged activation leading to neuroinflammatory responses through the released inflammatory factors. In turn, these pro-inflammatory cytokines contribute to the further neuritic dystrophy. NLD significantly suppressed the vicious inflammatory response in the hippocampus after intracerebroventricular injection of Aβ by protecting neurons against synaptic damage, reducing the production of CGA, and suppressing the activation and aggregation of microglia and the astrocytes. NLD also inhibits activation of the NF-κB signaling pathway and the ASC-dependent inflammasome (including NLRP3 in microglia, IPAF in astrocytes and NLRP1 in neurons) and associated caspase-1/IL-1β axis by lowering levels of pIκBα, nuclear p65, ASC and caspase 1 p20 following Aβ-infusion, resulting in reduced production of pro-inflammatory factors and thereby exerting anti-inflammatory effects.

This is the first study that establishes the vicious cycle of the neuroinflammatory network in AD and elucidates the neuroprotective mechanism of NLD in the rat AD model (Figure 8). NLD also reduced the CGA release from the Aβ deposits-induced neuritic dystrophy in AD, resulting in the reduction of activated microglia induced by CGA. Inhibition of NF-κB signaling pathway and ASC-dependent inflammasome/caspase-1/IL-1β axis by NLD diminished the overproduction of pro-inflammatory cytokines, potentially due to inhibition of activated microglia and astrocytes, thereby protecting neurons against neuritic dystrophy. Further, the downregulation of pro-inflammatory cytokines (TNF-α and IL-1β) mitigated the neuroinflammatory amplification in the astrocytes. Eventually, significantly reduced microglial and astrocyte activation, in addition to suppressed production of pro-inflammatory factors, contributed to the recovery of neuronal synapses and resulted in improved cognitive function.

This study has several limitations. Although the Aβ rat model of AD is widely used for investigating the neuroinflammation in AD [47, 48], the APP/PS1 transgenic rat model of AD is needed to ensure that our results are not due to impairments from mechanical injury in Aβ rat model of AD and not impacted by rat species variation. Moreover, NLD has a complex chemical composition and therefore the exact bioactive components of NLD that exert protective function against neuroinflammation in AD remain unclear. Furthermore, the mechanistic details of the effects of NLD that prevent activation of the ASC-dependent inflammasome ( NLRP3, IPAF and NLRP1) in the microglia, astrocytes and neurons is unknown and needs to be investigated. In addition, the protective mechanisms of NLD against neuritic dystrophy need to be studied in greater detail.

In summary, we demonstrate that NLD demonstrates significant neuroprotection against AD by improving cognitive function and helping recovery of the neural synapse by inhibiting the neuroinflammatory network between microglia, astrocytes and neurons. It inhibits both the NF-κB signaling pathway and the ASC-dependent inflammasome. Thus, our study demonstrates that NLD is a promising therapeutic agent for the treatment of AD.

## MATERIALS AND METHODS

### Preparation of NLD decoction

The NLD decoction was prepared from crude slices of *Herba epimedii* (Yinyanghuo), *Polygonum multiflorum* (Heshouwu), *Tortoise plastron* (Guiban), *Ossa draconis* (Longgu), *Polygala tenuifolia* (Yuanzhi), and *Rhizoma acori graminei* (shichangpu) in a 3:3:4:4:2:2 ratio. These herbs were obtained from the pharmacy of Second Xiangya Hospital, Central South University, Hunan Province, China. Each Chinese herbal piece was authenticated by Prof. SY Hu, Department of Chinese Herbal Medicine of Central South University. The voucher specimens (NO.20150268) were preserved in the Second Xiangya Hospital of Central South University (Changsha, China). The six constituents of NLD were soaked in distilled water (1:10 w/v) in a 3:3:4:4:2:2 ratio for 60 min at room temperature. Next, the decoctions were collected twice, first for 90 min in 1:10 w/v distilled water and second for 60 min in 1:8 w/v distilled water. Then, the NLD extractions were combined and lyophilized (16.2% w/w yield) according to standardized protocol [49]. The lyophilized powder was stored at 4°C.

### Liquid chromatography–mass spectrometry quadrapole time of flight (LC/MS-Q-TOF) analysis of 2,3,5,4′-tetrahydroxystilbene-2-O-β-D-glucopyranoside (THSG) and Icariin determination

The Agilent LC/MS-Q-TOF system (Santa Clara, CA, USA) was setup in the negative electrospray ionization (ESI) mode for chromatographic separation of THSG amd Icariin in an Agilent Technologies 1290 liquid chromatograph with an Cosmosil MS-II C18 column (250 × 4.6 mm, 5 μm). Solvent A was water containing 0.1% methanol and solvent B was acetonitrile. The gradient elution was started with 20%–30% B for 0–10 min followed by 30% B for 10-22 min, and finally 30%–33% B for 22–25 min at a flow rate of 1 ml/min. The column temperature was maintained at 25°C and the detection wavelength of the photodiode array detector was set at 270 nm. The injection volume was 10 μL in the full loop mode.

The exact mass of THFG and Icariin were determined using a Agilent 6520 Accurate-Mass Q-TOF LC/MS. The ESI operating parameters were as follows: drying gas (N_2_) temperature and flow rate: 320°C and 8L/min, respectively; nebulizer pressure: 35psig; sheath gas temperature: 320°C; sheath gas flow rate: 11L/min; capillary voltage: 3500V; nozzle voltage: 1000V. The TOFMS parameters were: fragmentor voltage: 110V; skimmer voltage: 65V; OCT 1 RF Vpp: 750V. These operations alongwith the data acquisition and analysis were controlled by Agilent Mass Hunter Workstation Software (B.04.00). The highly pure commercial Icariin and THSG ( purity > 98%, National Institute for the Control of Pharmaceutical and Biological Products, Beijing, China) were used as standards to confirm the retention time and mass spectrometry under the above mentioned conditions. For the analysis, 1.25 g of lyophilized NLD powder dissolved in 5 ml methanol and centrifuged at 8000 × g for 15 min at 4°C. The supernatant was filtered with a 0.22μm nylon filter and 10 μL of the sample was injected for LC/MS-Q-TOF analysis.

### Aβ_1-42_-infused rat model

The healthy male Sprague Dawley (SD) rats that weighed 200–250 g were obtained from the Laboratory Animal Research Center, Central South University and housed in a controlled breeding room with access to normal standard chow diet and tap water *ad libitum*. The room was maintained at 22–25°C, 12 h light/dark cycles and 50% ± 10% humidity. The rats were allowed to adapt for atleast 1 week before the experiments. First, the rats were fasted for 12 h. All animal experiments were conducted according to the guidelines approved by the Central South University Animal Ethics Committee.

The rats were injected intra-cerebroventricularly (ICV) with oligomeric Aβ_1–42_ to induce AD as established in our previous study [4], Briefly, rats were anesthetized with an intraperitoneal injection of 10% chloral hydrate (4 ml/kg) and then fixed on a stereotactic apparatus. The Aβ_1–42_ oligomers (5 μl, Sigma, St. Louis, MO, USA) were stereotaxically implanted into the bilateral cerebral ventricle using a stainless steel cannula. They were 1.1mm anterioposterior from bregma, 2.2 mm lateral to the sagittal suture, and 3.0 mm beneath the dura and fixed to the skull with dental cement.

### NLD treatment experimental design

Rats that underwent intracerebroventricular infusion were randomly distributed into five groups (*n* = 17 per group), namely, (1) sham rats that received bilateral ventricular injection of 0.9% NaCl without Aβ_1-42_ and same amount of normal saline by oral gavage every day; (2) vehicle rats that were given 0.9% NaCl intragastrically every day; (3) NLD-L rats that were orally administered 9 g/kg/day NLD; (4) NLD-M rats that were orally administered 27 g/kg/day NLD; and (5) NLD-H rats that were orally administered 54 g/kg/day NLD. Rats from each group then underwent Morris water maze (MWM) test from the 28th to 32nd day following which they were all anaesthetised with chloral hydrate. Then, brains from 3 rats from each group immediately preserved for Golgi-Cox staining. Also, 6 rats from each group were intracardially perfused with 0.9% NaCl followed by quickly fixing their brains in 4% paraformaldhyde. The rest of the 8 rats in each group were sacrificed by decapitation and their left hippocampus was frozen in liquid nitrogen and stored at −80°C for biochemical assays.

### Morris water maze test

The Morris water maze (MWM) performance of the rats from the five groups was analyzed to assess their cognitive function as described in our previous study [17]. Briefly, the rats were trained and subjected to four successive memory acquisition trials in a water maze to determine their ability to escape and find the platform. The measurements were performed every day between 28th and 32nd days after Aβ_1-42_ infusion. On the 32nd day, a spatial probe trial was performed to assess the spatial memory retention ability. Behavioral parameters were tracked and analyzed using the ANY-maze video tracking system (Stoelting Co., USA).

### Golgi-cox staining

To assess the failure of neural synapse in CA1 subregion of the hippocampus, Golgi-Cox staining was performed on brain samples of the five groups of rats using the FD Rapid Golgi Stain Kit (FD Neurotechnologies Inc. USA) as previously described [26]. In brief, the whole brain was immersed in Golgi-Cox solution (A + B) for 2 weeks at room temperature in the dark. Subsequently, the brains were transferred into solution C and stored at 4°C for 72 h in the dark. Then, 100μm thick coronal cryostat sections were obtained with the Cryotome (Thermo Electron Cooperation) at −22°C. After drying naturally at room temperature, the brain sections were stained with solution D + E for 15 min and dehydrated according to manufacturer's instructions. The dendrites and dendritic spines in CA1 were detected using an Olympus BX-51 microscope (Wetzlar, Germany). The pyramidal neurons with countable morphology in hippocampal CA1 region from each slide were selected for calculation (sham group: *n* = 25, vehicle group: *n* = 18, NLD-L group: *n* = 20, NLD-M group: *n* = 23, and NLD-H group: *n* = 21). The dendritic spine density was determined by a blinded analysis. Dendritic spines from three to four dendrites per neuron were counted for each sample. Dendritic spine density was determined as the spine number per dendritic length (dendritic spine density = spine numbers/dendritic length).

### Immunohistochemical analyses of Iba-1 and GFAP

The reactive microglia and astrocytes in the brain samples were visualized by immunohistochemistry (IHC) that was performed as described in the previous study with slight modifications [50]. Briefly, after fixation in 4% paraformaldhyde for 48 h, the brains were submerged in distilled water for 60 mins. Subsequently, they were dehydrated with an ethanol gradient from 60% to 100%. The dehydrated brains were then soaked in xylene for 30 mins and then embedded in paraffin at 65°C for 4 h. Then, serial 8 μm thick sections were cut and mounted on positively charged glass slides. The samples were then deparafinnized in xylene and then soaked in 80ml 10 mM sodium citrate buffer (pH 6.0) and microwaved at 95–98°C for 10 min for heat induced epitope retrieval. Then, the samples were treated with 3% H_2_O_2_ to block the endogenous peroxidases and with 1:60 horse serum for 30 min to block non-specific proteins. Finally, the sections were incubated with primary mouse anti-Iba-1 or anti-GFAP antibodies (1:400 in PBS) overnight at 4°C. Then, after washing, the primary antibodies were detected by biotinylated horse anti-mouse secondary antibodies and the avidin–biotin complex was developed according to the supplier's directions (Zymed Laboratories Inc.). One set of sections were also stained with hematoxylin for morphological examination Six to ten images per section were procured for analysis from each rat.

### Western blot protein analysis

Total protein lysate was prepared from 0.25 g hippocampal tissue with 500 μl RIPA lysis buffer (Applygen, Beijing) with protease inhibitors, and then centrifuged at12,000 rpm for 5 mins at 4°C to obtain the supernatants. Meanwhile, nuclear protein lysates were obtained using the NE-PER^®^ Nuclear and Cytoplasmic Extraction Reagents (Pierce Biotechnology, USA). Briefly, 0.25 g hippocampal tissue was homogenized with 500 μl CER and then centrifuged at 800 rpm for 5 min at 4°C. The cell precipitate was then dissolved with 500 μl NER and 80–100μl suspension buffer was added to obtain the nuclear protein lysates. The total protein concentration was measured using BCA method. The concentration of the protein samples was diluted to 4 mg/ml with lysis buffer and 5 × loading buffer. Then, 50 μg protein lysates were boiled for 5 mins and separated by SDS-PAGE followed by transfer to PVDF membrane. Then, the membranes were blocked by TBST buffer and incubated with one of the following primary antibodies: Chromogranin A (1:2000, Abcam, UK), Iba1 (1:1000, Wako, Japan), GFAP (1:1000, Dako, Japan), TNF-α (1:100, Santa cruz, USA), IL-1β (1:200, Proteintech, USA), IL-6 (1:500, Proteintech, USA), Phospho-IκBα (1: 1000, Cell Signaling Technology, USA), NF-κB p65 (1: 1000, Abcam, UK), ASC (1:1000, Proteintech, USA), Caspase-1 p20 (1:1000, Cell Signaling Technology, USA), PCNA (1:2000, Proteintech, USA) and β-actin (1:4000; Santa Cruz, USA). Next, the membranes were incubated with the horseradish peroxidase-coupled secondary antibody (KPL, 1:2000) and the protein bands were developed with an ECL kit (Themo) and visualized by exposures of various lengths to Kodak film. The bands were was expressed relative value to β-actin or PCNA.

### Quantitative real-time-PCR

Total RNA was extracted from hippocampal tissues and reverse transcribed into cDNA by SuperScript III Reverse Transcriptase (Invitrogen, Grand Island, NY, USA) according to protocol published in our previous study [4]. Quantitative real time PCR (Arraystar) was performed in the Applied Biosystems ViiA 7 Real-Time PCR System. The reaction mixture included cDNA and 2 × PCR Master Mix. The PCR amplification conditions were as follows: 95°C for 10 min, followed by 40 cycles of 95°C for 10 s and 60°C for 1 min. The relative amounts of mRNA were calculated using the 2^−ΔΔCt^ method and normalized to GAPDH. The primers used for this study are shown in Table 1.

**Table 1:** Sequences of PCR primers

Target gene	Forward primer (5′–3′)	Reverse primer (5′–3′)	Tm (°C)	Product length (bp)
ChromograninA	ATGCCTTTGAGGGAACCAC	GGGTATTGTTGGCTGTGTCC	59	117
TNF-α	GAAACACACGAGACGCTGAA	ATCCACTCAGGCATCGACAT	60	122
IL-1β	ACTTGGGCTGTCCAGATGAG	GTAGCTGCCACAGCTTCTCC	60	114
IL-6	CCAGTTGCCTTCTTGGGACT	GTCTGTTGTGGGTGGTATCCTCTGT	59	100
β-actin	CATCCTGCGTCTGGACCTGG	TAATGTCACGCACGATTTCC	60	116

Tm: annealing temperature.

### Statistical analysis

Data were expressed as mean ± SEM. Statistical analysis was performed with SPSS 17.0 software package and the plots were generated with Graphpad Prism software. Data for the escape latency of rats in the Morris water maze test were analyzed with two-way analysis of variance of repeated measures. The other data were analyzed with one-way ANOVA. *P* < 0.05 was considered significant.
